# Evaluation of a virulence gene repertoire as a means to investigate the role in the causal pathway to death of *Escherichia coli* isolated from postmortem specimens, northern Tanzania

**DOI:** 10.1093/ajcp/aqag088

**Published:** 2026-08-13

**Authors:** Deng B Madut, Matthew P Rubach, Cristina Costales, Robert J Rolfe, Manuela Carugati, Patrick T Amsi, Alex R Mremi, Nathan H Kalengo, Calvin Mosha, Annette Marandu, Michael J Maze, Blandina T Mmbaga, Kajiru G Kilonzo, Venance P Maro, Flemming Scheutz, John A Crump

**Affiliations:** Division of Infectious Diseases, Department of Medicine, Duke University, Durham, NC, United States; Division of Infectious Diseases, Department of Medicine, Duke University, Durham, NC, United States; Division of Infectious Diseases, Department of Medicine, Duke University, Durham, NC, United States; Division of Infectious Diseases, Department of Medicine, Duke University, Durham, NC, United States; Division of Infectious Diseases, Department of Medicine, Duke University, Durham, NC, United States; Kilimanjaro Christian Medical Centre, Moshi, Tanzania; Kilimanjaro Christian Medical Centre, Moshi, Tanzania; Kilimanjaro Christian Medical Centre, Moshi, Tanzania; Mawenzi Regional Referral Hospital, Moshi, Tanzania; Mawenzi Regional Referral Hospital, Moshi, Tanzania; Department of Medicine, University of Otago, Christchurch, New Zealand; School of Medicine, KCMC University, Moshi, Tanzania; School of Medicine, KCMC University, Moshi, Tanzania; School of Medicine, KCMC University, Moshi, Tanzania; The International Escherichia, Shigella, and Klebsiella Centre, Statens Serum Institut, Copenhagen, Denmark; Division of Infectious Diseases, Department of Medicine, Duke University, Durham, NC, United States; School of Medicine, KCMC University, Moshi, Tanzania; Centre for International Health, University of Otago, Dunedin, New Zealand

**Keywords:** Africa, autopsy, Tanzania, *Escherichia coli*, whole-genome sequencing

## Abstract

**Objectives:**

Interpreting postmortem microbiological data is challenging, especially when enteric organisms are isolated. We evaluated whether virulence gene profiles could distinguish hospitalized decedents in whom *Escherichia coli* was adjudicated to be in the causal pathway to death from those in whom it was not.

**Methods:**

We conducted a prospective autopsy study from 2016 through 2019 at 2 hospitals in northern Tanzania. Postmortem cultures were collected from multiple specimen types. The role of *E coli* in the causal pathway to death was adjudicated using clinical, microbiological, and histopathologic data. Separate from adjudication, whole-genome sequencing was performed on *E coli* isolates for classification as extraintestinal pathogenic *E coli* (ExPEC) vs non-ExPEC based on their virulence genes. Logistic regression assessed the odds of isolating ExPEC when *E coli* was adjudicated as being in the causal pathway to death.

**Results:**

We identified 74 *E coli* isolates from 57 (26.4%) of the 216 decedents enrolled in our study. Among decedents with *E coli* growth in culture, median (IQR) age was 38 (24-61) years, 14 (24.6%) were female, and *E coli* was adjudicated as being in the causal pathway to death in 17 (29.8%). Of these 17 decedents, 13 (76.5%) had at least 1 isolate designated as ExPEC compared with 24 (60.0%) of 40 decedents for whom *E coli* was not deemed causative of death. The odds of isolating ExPEC when *E coli* was in the causal pathway to death was 2.78 (95% CI, 0.62-12.3; *P* = .18).

**Conclusions:**

No single genomic marker, including ExPEC status, was sufficient to establish the causal role of *E coli* in death.

Key points

- Postmortem microbiology can help identify infectious causes of death, but interpreting organisms such as *E coli* is complicated by microbial translocation and potential contamination during autopsy.
- Genomic characterization, such as virulence gene profiling, offers a potential means to distinguish true infection from postmortem artifact.
- In this study, no genomic feature reliably predicted the role of *E coli* in the causal pathway to death, highlighting that the utility of genomic profiling for autopsy microbiology remains unclear.

## INTRODUCTION

Infectious diseases remain a leading cause of mortality in sub-Saharan Africa, but specific etiologies underlying these deaths remain poorly understood.^1^ This knowledge gap is partly due to the limited availability of autopsies, which are often restricted by shortages of trained personnel and inadequate infrastructure.^2^^,^^3^ By contrast, in some high-resource settings, autopsies have played a critical role in identifying infectious causes of death and generating insights that inform clinical care.^4^^,^^5^ Although the value of postmortem examination in uncovering infection-related etiologies is recognized,^6^ interpreting microbiological findings after death presents unique challenges.^7^^,^^8^

When microorganisms are isolated post mortem, several mechanisms may explain their presence.^7^^,^^8^ First, the organism may have invaded a target organ or body fluid during life and thus represents true infection. Second, postmortem decomposition can lead to microbial translocation across tissue planes, resulting in the presence of microorganisms in normally sterile sites post mortem.^7‐9^ This microbial translocation can be minimized if the body is maintained at 4 to 6 °C before the postmortem examination and if specimens are collected within 24 to 48 hours of death.^7^^,^^10^ Third, procedural and sample collection factors during autopsy, such as contamination from instruments or surfaces, may inadvertently introduce organisms into sterile spaces.^7^^,^^8^

The limitations in interpreting postmortem microbiology have been recognized for decades, but little progress has been made in improving approaches. To address this limitation, we propose a framework based on the genomic characterization of postmortem microbial isolates, with *Escherichia coli* serving as a model pathogen. Although the presence of *E coli* in postmortem specimens could represent translocation from heavily colonized body sites such as the gut or even contamination during sample collection, genomic studies have revealed that invasive disease is typically caused by specific subsets of strains termed extraintestinal pathogenic *E coli* (ExPEC).^11^ These strains can often be distinguished from other commensal and intestinal pathogenic *E coli* by their distinct virulence gene profiles.^12^^,^^13^ Further genomic analyses, including multilocus sequence typing (MLST), have shown that despite the broad diversity of *E coli* sequence types (STs), the majority of ExPEC infections are attributable to a relatively small number of STs.^14^ Similarly, despite more than 180 somatic antigen (O) groups, ExPEC strains cluster within a limited subset.^15^^,^^16^ Collectively, these distinctive genomic features of ExPEC strains provide a possible tool for interpreting the clinical relevance of *E coli* detected after death.

In the present study, we performed genomic characterization of *E coli* isolates from specimens obtained during postmortem examination of decedents at 2 hospitals in northern Tanzania. We then adjudicated the role of *E coli* in the causal pathway to death through a comprehensive review of each decedent’s clinical record, microbiological data, and pathologic findings. To assess whether genomic data could aid in interpreting postmortem microbiology, we compared the genomic characteristics of isolates implicated in death with those not implicated, with particular attention to their virulence gene profiles, STs, and O groups.

## METHODS

### Study setting

Participants were enrolled in a prospective cohort to understand the etiologies of febrile illness, including febrile deaths, among hospitalized patients at Kilimanjaro Christian Medical Centre (KCMC) and Mawenzi Regional Referral Hospital (MRRH) in Moshi, Tanzania. The study was conducted from September 2016 through May 2019, and some results have previously been published.^17‐20^ The KCMC is a 630-bed zonal referral hospital that serves the northern zone of Tanzania. The MRRH is a 300-bed regional hospital that serves the Kilimanjaro Region. Moshi (population 200 000) is the administrative center of the Kilimanjaro Region (population 1.6 million); regional HIV seroprevalence among individuals aged 15 to 49 years was 2.6% in 2016.^21^

### Study procedures

During the study period, all inpatient pediatric and adult medical deaths at KCMC and MRRH were screened for eligibility. We enrolled decedents with febrile illness, defined as an oral or tympanic temperature of 38.0 °C or higher within the first 48 hours of hospital admission or a reported history of fever within 72 hours before admission, as well as those without febrile illness. The target ratio of febrile to afebrile decedents was approximately 3:1.

Families of eligible decedents were offered a complete diagnostic autopsy (CDA) or, if the family refused the CDA, minimally invasive tissue sampling (MITS) post mortem.^22^^,^^23^ Decedents were enrolled following written informed authorization from their next of kin. Bodies were stored at 4 °C from the time of death until postmortem examination, which was targeted to occur within 24 hours of death. After enrollment, research personnel administered a survey to the next of kin to collect the decedent’s sociodemographic data. Data were abstracted from the medical record to understand each decedent’s clinical course. Postmortem examinations, either CDA or MITS, were performed by 1 of 3 study pathologists (A.R.M, P.T.A., C.C.). Biopsy needles were used to collect tissue specimens from the brain, heart, lung, liver, spleen, kidney, and bone marrow. For every decedent, standard microbiological sampling included the attempted collection of aerobic cultures from the subclavian vein, cerebrospinal fluid, liver, and bone marrow. Additionally, pathologists could collect cultures from any site that appeared abnormal on gross examination. Skin surfaces overlying sampling sites were disinfected with alcohol, followed by a povidone-iodine scrub before culture specimen collection.

### Microbiological methods

HIV antibody testing was performed on all decedents following authorization from the next of kin. HIV testing was performed on whole blood using the Standard Diagnostics Bioline HIV-1/HIV-2 3.0 test kit (Standard Diagnostics Inc, Kyonggi-do, South Korea) for screening, followed by the Unigold Rapid HIV test kit (Trinity Biotech, Bray, Ireland) for confirmation.

Blood cultures were collected in BacT/ALERT FA Plus bottles, loaded into the BacT/ALERT 3D microbial detection system, and incubated for 5 days. Other tissue and fluid samples were ground, if appropriate, and plated on blood, chocolate, and MacConkey agar plates. *E coli* biochemical identification was performed using API 20E (bioMérieux Inc, Marcy-I’Étoile, France) test strips. *E coli* isolates were stored at ‒70 °C and shipped on dry ice to the Statens Serum Institut in Copenhagen, Denmark, for genomic sequencing.

### Genomic methods

Genomic DNA was purified from *E coli* isolates using the DNeasy Blood & Tissue Kit (QIAGEN, Hilden, Germany). Sequencing was carried out on an Illumina MiSeq sequencer (Illumina, San Diego, CA, United States) using the Nextera XT Library preparation protocol for obtaining paired-end reads of 150 base pairs (bp) or 250 bp. Whole-genome sequencing data were preprocessed used a quality control pipeline (https://github.com/ssi-dk/SerumQC). Sequences were removed when there was contamination with more than 5% of non-*Escherichia* genera and if they represented isolates with genome sizes outside the range of 4.64 to 5.56 megabase pairs. Sequences were removed from the dataset if assemblies contained more than 350 contigs. *De novo* assembly was carried out using QIAGEN CLC Genomic Workbench. High-quality assemblies were selected for downstream analysis based on the following criteria: sequencing coverage greater than 30 times, minimum assembly length greater than 1 megabase pair, and contig lengths greater than 200 bp.

Assembled sequence data from *E coli* isolates were analyzed using typing tools from the Center for Genomic Epidemiology (CGE) (http://www.genomicepidemiology.org). The CGE contains databases for *in silico* MLST, serotyping (SerotypeFinder), virulence analysis (VirulenceFinder), and antimicrobial resistance prediction. The various CGE Finder tools used a Basic Local Alignment Search Tool–based approach to detect the genes of interest in the *de novo* assembled genome and subsequently identify them against the appropriate reference database. Detection parameters were as follows: for VirulenceFinder and SerotypeFinder, 85% sequence identity and 60% sequence coverage; for ResFinder, 90% sequence identity and 60% sequence coverage; and for MLSTFinder, using the 7 loci (*adk*, *gyrB*, *fumC*, *icd*, *mdh*, *purA*, and *recA*) scheme. Conventional serotyping was employed if the results of *in silico* serotyping were ambiguous.^24^

### Definitions

In a living person, *E coli* may be classified as ExPEC based on isolation from a normally sterile site. Bacterial translocation or contamination during autopsy, however, may render this definition unreliable for postmortem microbiology. Consequently, we used the genotypic definition, classifying *E coli* isolates as ExPEC if they were positive for 2 or more of the following virulence genes: *pap, sfa/foc, afa/dra, iutA,* or *kpsMT II*.^13^^,^^25^ In addition, isolates were classified as uropathogenic *E coli* (UPEC) if they were positive for at least 3 of the following genes: *chuA*, *fyuA*, *vat*, or *yfcV*.^13^^,^^25^

### Adjudication process

Assessment of the role of *E coli* in the causal pathway to death was made by 2 physician adjudicators (D.B.M., R.J.R.). *E coli* was considered to be in the causal pathway if it was the attributed pathogen in a condition directly leading to death, as outlined in standard death certificate guidance for identifying both immediate and underlying causes of death.^26^ To facilitate a standardized and reproducible assessment, a point-based scoring system was developed that integrated information from antemortem clinical findings, postmortem microbiology, and postmortem gross and histopathologic findings (see **Supplemental Material**). This scoring system was informed by the clinical expertise of the study investigators and existing research on the interpretation of postmortem microbiology.^7^^,^^10^ The score was used to classify the role of *E coli* as unlikely or possible in the causal pathway to death. After independent adjudication, the reviewers convened to resolve any discrepancies. If consensus could not be reached, a third adjudicator (M.P.R.) served as the tiebreaker. All adjudicators were blinded to the genomic data of isolates.

### Analysis

Statistical analyses were performed using STATA, version 19.5, statistical software (Stata Corporation LLC, College Station, TX, United States) and R, version 4.3.2, software. Continuous variables were expressed using medians and IQRs; categorical variables were expressed as frequencies. Data were evaluated at multiple hierarchical levels. First, variables summarized at the decedent level were denoted as such, with the term *decedent* used when referring to analyses aggregated by individual. Second, variables summarized at the specimen-site level referred to distinct anatomical sites from which *E coli* was recovered. If *E coli* was isolated more than once from the same site within a decedent (e.g., multiple positive blood cultures), that site was counted once. Finally, among decedents with *E coli* recovered from multiple specimen sites, we enumerated genomically distinct isolates. Isolates were classified as distinct if they differed by ST. When isolates from multiple specimen sites shared the same ST, they were further differentiated as distinct if they expressed different serotypes.

Descriptive analyses were used to evaluate the distribution of STs, O groups, and antimicrobial resistance genes among distinct isolates classified as ExPEC vs non-ExPEC. Similar comparisons were made between distinct isolates from decedents for whom *E coli* was determined to be in the causal pathway to death and those in whom it was not.

To further contextualize the STs of distinct isolates identified in our cohort, we assessed whether the STs were among the 20 most commonly reported ExPEC STs in global datasets, as described in a meta-analysis by Manges  et al.^14^ These globally prevalent STs, listed in descending order of frequency, are ST131, ST69, ST10, ST405, ST38, ST95, ST648, ST73, ST410, ST393, ST354, ST12, ST127, ST167, ST58, ST617, ST88, ST23, ST117, and ST1193. We also assessed whether the O groups identified among distinct isolates matched the most prevalent O groups, as described by Weerdenburg  et al^16^ and defined as those found in 1% or more of global isolates. Collectively, these O groups account for approximately 80% of the O groups reported among patients with invasive *E coli* infections in global datasets and include O25, O2, O6, O1, O75, O15, O8, O16, O4, O18, the O77 group, O153, O9, O101/O162, O86, and O13.

Finally, multivariable logistic regression models, adjusted for age and sex, were constructed to evaluate the odds of isolating at least 1 ExPEC isolate from a decedent when *E coli* was adjudicated as being in the causal pathway to death.

### Ethics statement

Ethics approval was obtained from the Research Ethics Committee of Kilimanjaro Christian Medical University College (reference 295), the National Health Research Ethics Coordinating Committee of the Tanzania National Institute for Medical Research (reference NIMR/HQ/R.8c/Vol.IX/1000), and an institutional review board of Duke University (Durham, NC, United States; reference Pro00016134). Written authorization was obtained from the next of kin for all decedents.

## RESULTS

### Characteristics of decedents

**Figure 1:**
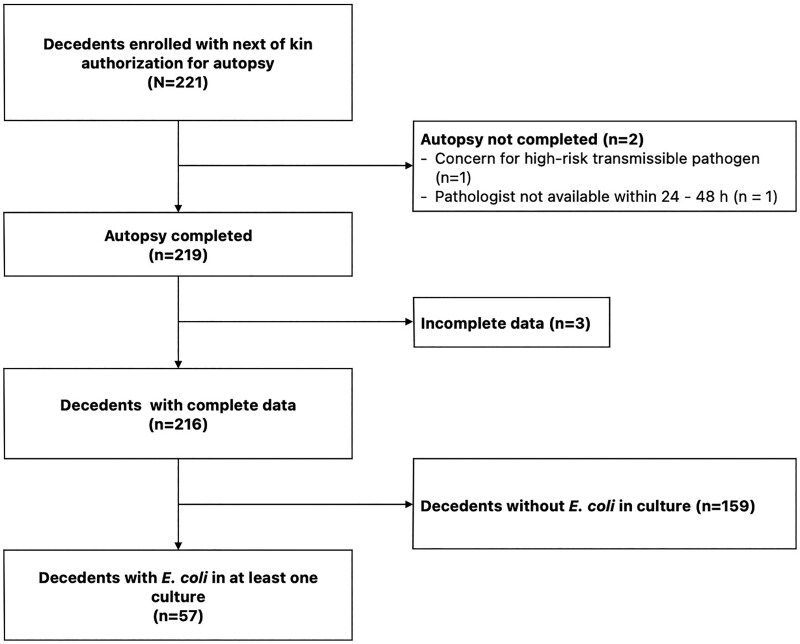
Flow diagram of decedents enrolled in a postmortem study in northern Tanzania, 2016-2019. E coli indicates *Escherichia coli*.

Of 216 decedents in our cohort, 57 (26.4%) had at least 1 culture that yielded *E coli* (**Figure 1**). Among the 57 decedents who had at least 1 culture that yielded *E coli*, the median (IQR) age was 38 (24-61) years, 14 (24.6%) were female, and 11 (19.3%) were HIV seropositive. A MITS was performed in 13 (22.8%) decedents, and 44 (77.2%) decedents underwent CDA. The median (IQR) time from death to autopsy was 21.3 (11.5-30.6) hours. Characteristics of decedents with *E coli* in postmortem cultures are shown in **Table 1**, and characteristics of the full cohort are provided in **Table S1**.

**Table 1:** Demographic and Clinical Characteristics Among Decedents With *Escherichia coli* Isolated From Postmortem Culture, Northern Tanzania, 2016-2019

		*E coli* adjudicated as being in the causal pathway to death	
Variable	**Overall (*n*** = **57)**	**Unlikely (*n*** = **40)**	**Possible (*n*** = **17)**
Age, median (IQR), y	38 (24-61)	39 (23-63)	37 (26-54)
Sex, No. (%)			
Male	42 (73.6)	32 (80.0)	10 (58.8)
Female	14 (24.6)	8 (20.0)	6 (35.3)
Missing	1	0	1
Status at enrollment, No. (%)			
Afebrile	13 (22.8)	10 (25.0)	3 (17.6)
Febrile	44 (77.2)	30 (75.0)	14 (82.4)
Autopsy type			
MITS	13 (22.8)	9 (22.5)	4 (23.5)
CDA	44 (77.2)	31 (77.5)	13 (76.5)
Time from admission to death, median (IQR), d	3.9 (1.2-8.7)	3.8 (1.8-8.2)	4.4 (1.2-9.9)
Time to autopsy, median (IQR), h	21.3 (11.5-30.6)	19.4 (11.6-27.3)	26.9 (10.9-34.1)
HIV serostatus, No. (%)			
Negative	46 (80.7)	32 (80.0)	14 (82.4)
Positive	11 (19.3)	8 (20.0)	3 (17.6)
Abdominal pain, No. (%)			
No	24 (42.1)	18 (45.0)	6 (35.3)
Yes	20 (35.1)	13 (32.5)	7 (41.2)
Unknown	10 (17.5)	6 (15.0)	4 (23.5)
Missing	3	3	0
Dysuria, No. (%)			
No	37 (64.9)	28 (70.0)	9 (52.9)
Yes	5 (8.8)	3 (7.5)	2 (11.8)
Unknown	12 (21.1)	6 (15.0)	6 (35.3)
Missing	3	3	0
≥1 ExPEC isolate, No. (%)			
No	20 (35.1)	16 (40.0)	4 (23.5)
Yes	37 (64.9)	24 (60.0)	13 (76.5)
Distinct isolate, No. (%)			
1	44 (77.2)	33 (82.5)	11 (64.7)
>1	13 (22.8)	7 (17.5)	6 (35.3)
Specimen sites with *E coli* culture growth, No. (%)			
1	39 (68.4)	34 (85.0)	5 (29.4)
2-4	13 (22.8)	4 (10.0)	9 (52.9)
>4	5 (8.8)	2 (5.0)	3 (17.6)

Abbreviations: CDA, complete diagnostic autopsy; ExPEC, extraintestinal pathogenic *Escherichia coli*; MITS, minimally invasive tissue sampling.

### Characteristics of *E coli* isolates

Among the 57 decedents with *E coli* isolated from postmortem cultures, a total of 121 specimen sites yielded *E coli* (**Table 2**). When comparing specimen sites that yielded ExPEC and non-ExPEC isolates (**Table 2**), ExPEC isolates were more frequently recovered from blood (55.3% vs 44.7%), bone marrow (66.7% vs 33.3%), liver (65.2% vs 34.8%), and peritoneal fluid (57.9% vs 42.1%). In contrast, non-ExPEC isolates were more common in cerebrospinal fluid (80.0% vs 20.0%), pericardial fluid (62.5% vs 37.5%), and pleural fluid (55.6% vs 44.4%).

**Table 2:** Specimen Site of Culture Collection and ExPEC Virulence Gene Status of 121 Postmortem *Escherichia coli* Isolates

		ExPEC status	
Variable	No.	**Not ExPEC (*n*** = **55)**	**ExPEC (*n*** = **66)**
Specimen site of culture collection			
Blood	38	17 (44.7)	21 (55.3)
Bone marrow	12	4 (33.3)	8 (66.7)
Brain tissue	1	0 (0)	1 (100)
Cerebrospinal fluid	10	8 (80.0)	2 (20.0)
Kidney tissue	1	0 (0)	1 (100)
Liver tissue	23	8 (34.8)	15 (65.2)
Pericardial fluid	8	5 (62.5)	3 (37.5)
Peritoneal fluid	19	8 (42.1)	11 (57.9)
Pleural fluid	9	5 (55.6)	4 (44.4)

Abbreviation: ExPEC, extraintestinal pathogenic *Escherichia coli.*

**Figure 2:**
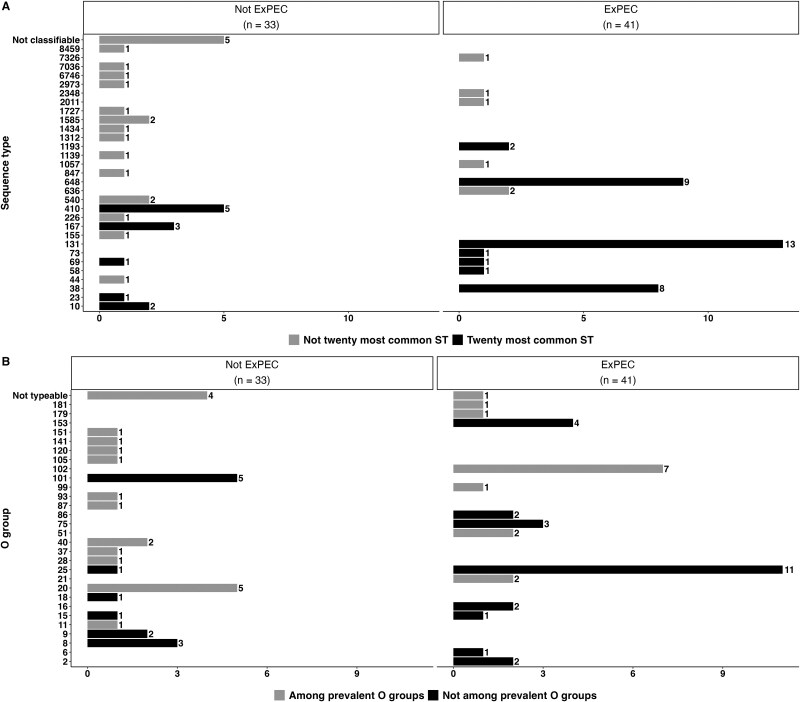
Extraintestinal pathogenic *Escherichia coli* (ExPEC) status, sequence type (ST), and somatic antigen (O) group of 74 *E coli* isolates from decedents, northern Tanzania, 2016-2019. (**A**) Frequency of STs and (**B**) O groups, by ExPEC status. (**A**) Sequence types among the 20 most frequently reported STs in global data, as reported by Manges  et al,^14^ are presented as black bars. (**B**) O groups among the most prevalent O groups in global datasets, as reported by Weerdenburg  et al,^16^ are presented as black bars.

Among the 121 cultures that yielded *E coli*, we identified 74 distinct *E coli* isolates. Of distinct isolates, 41 (55.4%) met ExPEC criteria based on their virulence gene profile. Among these, 25 (61.0%) also met virulence gene criteria for UPEC (**Table S2**). The 41 ExPEC isolates comprised 12 STs, with 13 (31.7%) being ST131 (**Figure 2**). The STs of 35 (85.4%) ExPEC isolates were among the 20 most frequently reported by Manges  et al.^14^ We identified 15 O groups among the ExPEC isolates, with 11 (26.8%) being O25 (**Figure 2**). The O groups of 26 (63.4%) ExPEC isolates were among the most prevalent O groups in global datasets, as described by Weerdenburg  et al.^16^ The distribution of antibacterial resistance genes possessed by ExPEC isolates is detailed in **Table S3**.

Of the 74 distinct *E coli* isolates, 33 (44.6%) were not classified as ExPEC; of these, 1 (3.0%) met virulence gene criteria for UPEC. The 33 non-ExPEC isolates comprised 20 STs, with 5 (15.2%) being ST410 (**Figure 2**). Of non-ExPEC isolates, 12 (36.4%) were among the 20 most frequently reported in global datasets.^14^ We identified 18 O groups among the non-ExPEC isolates, with O20 and O101 accounting for 5 (15.2%) each. Of non-ExPEC isolates, 13 (39.4%) belonged to globally prevalent O groups (**Figure 2**).^16^ The distribution of antibacterial resistance genes the non-ExPEC isolates possessed is detailed in **Table S3**.

### Adjudication of *E coli* in the causal pathway to death

**Figure 3:**
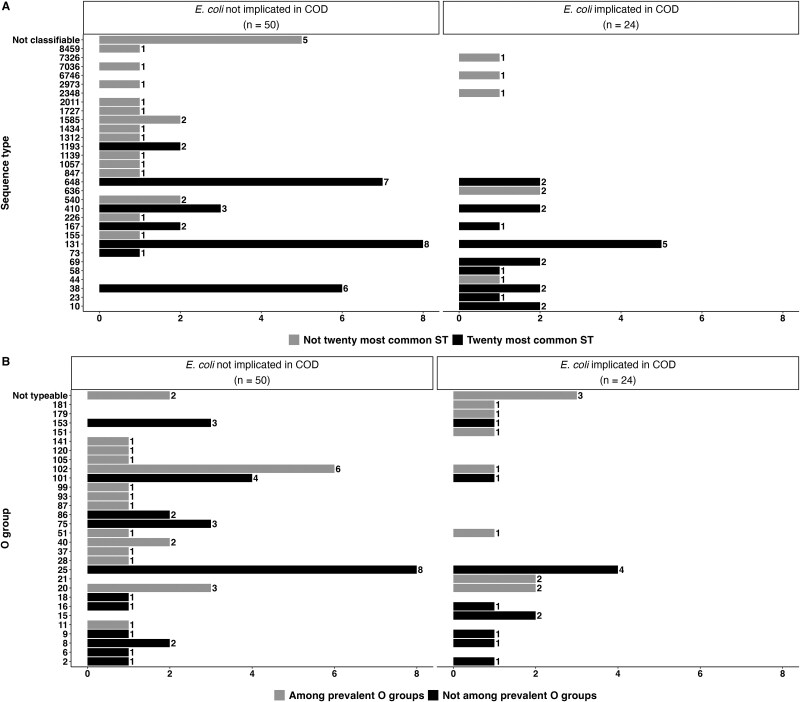
Adjudication of the role of *Escherichia coli* in the causal pathway to death, ST, and O group of 74 *E coli* isolates from decedents, northern Tanzania, 2016-2019. (**A**) Frequency of STs and (**B**) O groups, by adjudication status. (**A**) Sequence types among the 20 most frequently reported STs in global data, as reported by Manges  et al,^14^ are presented as black bars. (**B**) O groups among the most prevalent O groups in global datasets, as reported by Weerdenburg  et al,^16^ are presented as black bars. COD indicates cause of death; O, somatic antigen; ST, sequence type.

Among the 57 decedents with at least 1 culture that yielded an *E coli* isolate, *E coli* was adjudicated as in the causal pathway to death in 17 (29.8%) (**Table 1**). Of these 17 decedents, there were 24 distinct isolates, of which 15 (62.5%) were ExPEC. In comparison, among the 40 decedents in whom *E coli* was adjudicated not to be in the causal pathway to death, there were 50 distinct isolates, of which 26 (52.0%) were ExPEC isolates. Isolate characteristics by adjudication status are shown in **Figure 3** and **Table S3**.

Among the 17 decedents with *E coli* in the causal pathway, 13 (76.5%) had at least 1 isolate that was categorized as ExPEC (**Table 1**). In comparison, among the 40 decedents for whom *E coli* was deemed not to be in the causal pathway to death, 24 (60.0%) had at least 1 isolate that was categorized as ExPEC. The odds of isolating an ExPEC from a decedent when *E coli* was adjudicated as being in the causal pathway to death was 2.78 (95% CI, 0.63-12.3; *P* = .18).

## DISCUSSION

We evaluated the virulence gene profiles of *E coli* isolated post mortem to assess the relationship between ExPEC status and their adjudicated role in the causal pathway to death. Although a high proportion of isolates from decedents in whom *E coli* was adjudicated to have a causal role in death met ExPEC virulence gene criteria, ExPEC isolates were also frequently isolated from decedents in whom *E coli* was not implicated in the cause of death. Our findings underscore the challenges of interpreting postmortem microbiological data and suggest that ExPEC status alone may not be a reliable genomic marker for establishing the causal role of *E coli* in death.

We observed expected differences between ExPEC and non-ExPEC isolates across several genomic characteristics. Notably, the distributions of STs, O groups, and resistance genes differed between the 2 groups. Differences were also noted in the proportion of ExPEC and non-ExPEC isolates recovered from various specimen sites, although these patterns were difficult to interpret. Despite these anticipated differences, 2 findings ran counter to our expectations. First, although we anticipated that ExPEC strains would frequently be identified in decedents in whom *E coli* contributed to death, the proportion was lower than expected. For example, in our prior study of antemortem bloodstream infections among hospitalized patients in northern Tanzania, 91% of *E coli* isolates met ExPEC virulence gene criteria.^20^ By comparison, in our present analysis, 62.5% of *E coli* isolates met ExPEC virulence gene criteria among decedents for whom *E coli* was adjudicated as having a causal role in death. Second, our regression analysis demonstrated that ExPEC status was not associated with a causal role of *E coli* in death, partly because ExPEC strains were also recovered from decedents in whom *E coli* was not adjudicated to be causal in death.

The frequent recovery of ExPEC isolates from decedents in whom *E coli* was not adjudicated to have contributed to death warrants further discussion. Broadly, it is important to recognize that *E coli* was cultured from at least 1 sample in 57 of 216 decedents yet was deemed to be in the causal pathway to death in only 17 of these 57 decedents. This finding suggests that many isolates likely reflected postmortem translocation or contamination. Although we attempted to limit these processes by maintaining bodies at 4 °C immediately after death, conducting the postmortem examination within 24 hours after death, and using sterile techniques for sample collection, translocation and contamination cannot be excluded. To this end, a plausible hypothesis for the high proportion of ExPEC isolates, even among decedents in whom *E coli* was not considered causal in death, is that contamination or translocation disproportionately involved ExPEC strains, possibly because the same virulence traits that enhance survival, dissemination, or persistence in extraintestinal sites during life play a similar role after death.

Our study has several limitations. First, decedents were enrolled from only 2 hospitals in northern Tanzania; thus, our findings may not be generalizable to other regions of Tanzania or other countries in sub-Saharan Africa. Second, end point adjudications are inherently susceptible to misclassification.^27^ Although we implemented a structured adjudication process, with a focus on consistency and reproducibility, the extent of misclassification is difficult to quantify. Third, genomic characterization of *E coli* isolates from Tanzania—and, more broadly, sub-Saharan Africa—remains limited.^28^^,^^29^ As a result, comparisons between our isolates and global datasets are constrained by the scarcity of regional genomic data. Finally, it is important to recognize that even during life, not all *E coli* isolates recovered from normally sterile sites meet ExPEC criteria based on their virulence gene repertoire. Although ExPEC-associated genes are enriched among isolates causing invasive disease, *E coli* strains that lack the canonical ExPEC virulence profile have also been documented in such infections.^20^^,^^30^^,^^31^ This nuance should be borne in mind when interpreting our findings and, more broadly, when evaluating genomic data describing *E coli* associated with extraintestinal disease.

In summary, we observed that *E coli* was frequently isolated post mortem in decedents in northern Tanzania. The frequent detection of *E coli* among decedents in whom it was not adjudicated to have a causal role in death suggests that postmortem translocation or contamination may have been common. Moreover, no single genomic marker, including ExPEC status, was sufficient to establish the causal role of *E coli* in death. Taken together, our findings underscore the persistent challenges in interpreting postmortem microbiological data and raise the question of whether genomic profiling can improve interpretation of those data.

## Supplementary Material

aqag088_Supplementary_Data

## Data Availability

Data collected for the study include individual participant data and a data dictionary defining each field in the set. Data will be made available for researchers who can meet the criteria for accessing sensitive and confidential data, including obtaining ethics committee review and complying with data management and transfer agreements. Requests may be sent to the corresponding author (deng.madut@duke.edu). Raw sequencing reads of *E coli* isolates have been deposited in the National Center for Biotechnology Information Sequence Read Archive under BioProject PRJNA1353880 (submission identifier SUB15731818), with accession numbers SAMN52936960 through SAMN52937080.
