# Supplementary material for: Evaluation of a virulence gene repertoire as a means to investigate the role in the causal pathway to death of Escherichia coli isolated from postmortem specimens, northern Tanzania
Source: Am J Clin Pathol. 2026 Aug 13;166(2):aqag088. doi: 10.1093/ajcp/aqag088 (PMC13472684; doi:10.1093/ajcp/aqag088)
Supplement: Supplementary file 1 [file aqag088_supplementary_data.docx]

**SUPPLEMENTARY INFORMATION**

Madut DB, et al.^[1]^*

**Evaluation of virulence gene repertoire as a means to investigate the role in the causal pathway to death of *Escherichia coli* isolated from postmortem specimens, northern Tanzania**

Table of Contents

[Adjudication metrics	2](#_Toc212614746)

[Supplementary Table 1. Demographic and clinical characteristics among decedents enrolled in a postmortem study, northern Tanzania, 2016-2019	3](#_Toc212614747)

[Supplementary Table 2. Characteristics of 74 *E. coli* isolates stratified by their possession of ExPEC virulence genes among decedents enrolled in a postmortem study, northern Tanzania, 2016-2019	5](#_Toc212614748)

[Supplementary Table 3. Characteristics of 74 *E. coli* isolates stratified by their causal role in the pathway to death among decedents enrolled in a postmortem study, northern Tanzania, 2016-2019	7](#_Toc212614749)

[References	10](#_Toc212614750)

Adjudication metrics. Metrics for adjudicating the role of *E. coli* in the causal pathway to death among decedents in northern Tanzania

| Antemortem clinical findings | |
| --- | --- |
| Points | Criteria |
| 0 | Clinical history not consistent with infection |
| 1 | Infectious etiology possible but history not specific for *E. coli* infection |
| 2 | History could be consistent with *E. coli* (e.g. pyelonephritis, intra-abdominal pathology) |
| 3 | *E. coli* infection confirmed in the antemortem setting (e.g. culture positive) |

| Post-mortem microbiology | |
| --- | --- |
| Points | Criteria |
| 1 | *E. coli* isolated in one culture, either alone or with other organisms |
| 2 | *E. coli* isolated from multiple cultures with only one other organism |
| 3 | Pure growth of *E. coli* from at least two cultures |

| Autopsy | |
| --- | --- |
| Points | Criteria |
| 0 | No significant histologic findings or abnormal but another etiology more apparent |
| 1 | Abnormal histologic finding that could represent *E. coli* infection |
| 2 | Autopsy abnormalities strongly correlate with *E. coli* (e.g. pyelo) |
| 3 | *E. coli* confirmed on staining |

| Score | Adjudication |
| --- | --- |
| 1-4 | Unlikely |
| 5-9 | Possible |

| Supplementary Table 1. Demographic and clinical characteristics among decedents enrolled in a postmortem study, northern Tanzania, 2016-2019 | | | |
| --- | --- | --- | --- |
|  |  | ***E. coli* status in culture** | |
| **Variables** | **Overall (N=216)** | ***E. coli* not isolated (N=159)** | ***E. coli* isolated (N=57)** |
| **Age in years, median (IQR)** | 45 (24.8 - 65.6) | 46 (25.2 - 66) | 38 (24 - 61) |
| **Sex** |  |  |  |
| Male | 137 (63.7%) | 95 (59.7%) | 42 (75.0%) |
| Female | 78 (36.3%) | 64 (40.3%) | 14 (25.0%) |
| Missing | 1 | 0 | 1 |
| **Enrollment status** |  |  |  |
| Afebrile | 53 (24.5%) | 40 (25.2%) | 13 (22.8%) |
| Febrile | 163 (75.5%) | 119 (74.8%) | 44 (77.2%) |
| **Autopsy type** |  |  |  |
| MITS | 37 (17.1%) | 24 (15.1%) | 13 (22.8%) |
| CDA | 179 (82.9%) | 135 (84.9%) | 44 (77.2%) |
| **Time from admission to death, days, median (IQR)** | 2.6 (1.0 - 6.6) | 2.2 (0.9 - 5.4) | 3.9 (1.2 - 8.7) |
| **Time to autopsy, hours, median (IQR)** | 22.5 (14.0 - 31.7) | 23.2 (14.2 - 32.5) | 21.3 (11.5 - 30.6) |
| **HIV serostatus** |  |  |  |
| Negative | 162 (76.1%) | 116 (74.4%) | 46 (80.7%) |
| Positive | 51 (23.9%) | 40 (25.6%) | 11 (19.3%) |
| Missing | 3 | 3 | 0 |
| **Abdominal pain** |  |  |  |
| No | 111 (54.4%) | 87 (58.0%) | 24 (44.4%) |
| Yes | 53 (26.0%) | 33 (22.0%) | 20 (37.0%) |
| Unknown | 40 (19.6%) | 30 (20.0%) | 10 (18.5%) |
| Missing | 12 | 9 | 3 |
| **Dysuria** |  |  |  |
| No | 142 (69.6%) | 105 (70.0%) | 37 (68.5%) |
| Yes | 15 (7.4%) | 10 (6.7%) | 5 (9.3%) |
| Unknown | 47 (23.0%) | 35 (23.3%) | 12 (22.2%) |
| Missing | 12 | 9 | 3 |

Data presented as n (%) unless otherwise specified

Abbreviation: IQR, interquartile range; MITS, minimally invasive tissue sampling; CDA, complete diagnostic autopsy

| Supplementary Table 2. Characteristics of 74 *E. coli* isolates stratified by their possession of ExPEC virulence genes among decedents enrolled in a postmortem study, northern Tanzania, 2016-2019 | | | |
| --- | --- | --- | --- |
|  |  | **ExPEC status** | |
| **Variables** | **Overall (N=74)** | **Not ExPEC (N=33)** | **ExPEC (N=41)** |
| **UPEC** |  |  |  |
| No | 48 (64.9%) | 32 (97.0%) | 16 (39.0%) |
| Yes | 26 (35.1%) | 1 (3.0%) | 25 (61.0%) |
| **ST among twenty most common (1)** |  |  |  |
| No | 27 (36.5%) | 21 (63.6%) | 6 (14.6%) |
| Yes | 47 (63.5%) | 12 (36.4%) | 35 (85.4%) |
| **O group among most prevalent (2)** |  |  |  |
| No | 35 (47.3%) | 20 (60.6%) | 15 (36.6%) |
| Yes | 39 (52.7%) | 13 (39.4%) | 26 (63.4%) |
| **Beta-lactamase gene detected** |  |  |  |
| No | 25 (33.8%) | 15 (45.5%) | 10 (24.4%) |
| Yes | 49 (66.2%) | 18 (54.5%) | 31 (75.6%) |
| **Tetracycline resistance gene** |  |  |  |
| No | 29 (39.2%) | 14 (42.4%) | 15 (36.6%) |
| Yes | 45 (60.8%) | 19 (57.6%) | 26 (63.4%) |
| **Sulfonamide resistance gene** |  |  |  |
| No | 26 (35.1%) | 13 (39.4%) | 13 (31.7%) |
| Yes | 48 (64.9%) | 20 (60.6%) | 28 (68.3%) |
| **Fluoroquinolone resistance gene** |  |  |  |
| No | 47 (63.5%) | 20 (60.6%) | 27 (65.9%) |
| Yes | 27 (36.5%) | 13 (39.4%) | 14 (34.1%) |
| **Aminoglycoside resistance gene** |  |  |  |
| No | 25 (33.8%) | 12 (36.4%) | 13 (31.7%) |
| Yes | 49 (66.2%) | 21 (63.6%) | 28 (68.3%) |
| **Macrolide resistance gene** |  |  |  |
| No | 49 (66.2%) | 25 (75.8%) | 24 (58.5%) |
| Yes | 25 (33.8%) | 8 (24.2%) | 17 (41.5%) |
| **Trimethoprim resistance gene** |  |  |  |
| No | 24 (32.4%) | 13 (39.4%) | 11 (26.8%) |
| Yes | 50 (67.6%) | 20 (60.6%) | 30 (73.2%) |

Data presented as n (%) unless otherwise specified

Abbreviation: ExPEC, extraintestinal pathogenic *E.* coli; UPEC, uropathogenic *E. coli*; ST, sequence type

| Supplementary Table 3. Characteristics of 74 *E. coli* isolates stratified by their causal role in the pathway to death among decedents enrolled in a postmortem study, northern Tanzania, 2016-2019 | | | |
| --- | --- | --- | --- |
|  |  | ***E. coli* implicated in causal pathway to death** | |
| **Variables** | **Overall (N=74)** | **Unlikely (N=50)** | **Possible (N=24)** |
| **ExPEC status** |  |  |  |
| Not ExPEC | 33 (44.6%) | 24 (48.0%) | 9 (37.5%) |
| ExPEC | 41 (55.4%) | 26 (52.0%) | 15 (62.5%) |
| **UPEC** |  |  |  |
| No | 48 (64.9%) | 32 (64.0%) | 16 (66.7%) |
| Yes | 26 (35.1%) | 18 (36.0%) | 8 (33.3%) |
| **ST among twenty most common (1)** |  |  |  |
| No | 27 (36.5%) | 21 (42.0%) | 6 (25.0%) |
| Yes | 47 (63.5%) | 29 (58.0%) | 18 (75.0%) |
| **O group among most prevalent (2)** |  |  |  |
| No | 35 (47.3%) | 23 (46.0%) | 12 (50.0%) |
| Yes | 39 (52.7%) | 27 (54.0%) | 12 (50.0%) |
| **Beta-lactamase gene detected** |  |  |  |
| No | 25 (33.8%) | 18 (36.0%) | 7 (29.2%) |
| Yes | 49 (66.2%) | 32 (64.0%) | 17 (70.8%) |
| **Tetracycline resistance gene** |  |  |  |
| No | 29 (39.2%) | 22 (44.0%) | 7 (29.2%) |
| Yes | 45 (60.8%) | 28 (56.0%) | 17 (70.8%) |
| **Sulfonamide resistance gene** |  |  |  |
| No | 26 (35.1%) | 19 (38.0%) | 7 (29.2%) |
| Yes | 48 (64.9%) | 31 (62.0%) | 17 (70.8%) |
| **Fluoroquinolone resistance gene** |  |  |  |
| No | 47 (63.5%) | 32 (64.0%) | 15 (62.5%) |
| Yes | 27 (36.5%) | 18 (36.0%) | 9 (37.5%) |
| **Aminoglycoside resistance gene** |  |  |  |
| No | 25 (33.8%) | 18 (36.0%) | 7 (29.2%) |
| Yes | 49 (66.2%) | 32 (64.0%) | 17 (70.8%) |
| **Macrolide resistance gene** |  |  |  |
| No | 49 (66.2%) | 32 (64.0%) | 17 (70.8%) |
| Yes | 25 (33.8%) | 18 (36.0%) | 7 (29.2%) |
| **Trimethoprim resistance gene** |  |  |  |
| No | 24 (32.4%) | 19 (38.0%) | 5 (20.8%) |
| Yes | 50 (67.6%) | 31 (62.0%) | 19 (79.2%) |

Data presented as n (%)

Abbreviation: ExPEC, extraintestinal pathogenic *E.* coli; UPEC, Uropathogenic *E. coli*; ST, sequence type

References**:**

1.	Manges AR, Geum HM, Guo A, Edens TJ, Fibke CD, Pitout JDD. 2019. Global Extraintestinal Pathogenic Escherichia coli (ExPEC) Lineages. Clin Microbiol Rev 32:e00135-18.

2.	Weerdenburg E, Davies T, Morrow B, Zomer AL, Hermans P, Go O, Spiessens B, van den Hoven T, van Geet G, Aitabi M, DebRoy C, Dudley EG, Bonten M, Poolman J, Geurtsen J. 2023. Global Distribution of O Serotypes and Antibiotic Resistance in Extraintestinal Pathogenic Escherichia coli Collected From the Blood of Patients With Bacteremia Across Multiple Surveillance Studies. 3. Clin Infect Dis 76:e1236–e1243.

1. * Correspondence to Deng Madut, Duke University; Durham, North Carolina, United States; [deng.madut@duke.edu](mailto:deng.madut@duke.edu)
